# Operation on the Closure of the External Orifice of the Vaginal Canal

**Published:** 1846-10

**Authors:** Alfred E. Ames

**Affiliations:** Roscoe, Illinois


ARTICLE III.

*Operation on the Closure of the External Orifice of the Vaginal Canal.* By ALFRED E. AMES, M. D., Roscoe, Illinois.

I was called, May 6, 1846, to see a female child, æt. 93 days, who had a perfect closure of the vaginal canal, at the roots of the labia majora and minora, below the meatus urinarius.

On inquiry, the mother informed me that she had just noticed the closure, said the child had always been well, and that there had been no inflammation about the genital organs since the child was born. The body of the meatus urinarius was rather more prominent than natural; no difficulty in voiding urine.

To divide the membrane, we had the labia majora and minora separated, then with the sharp-pointed bistoury made an incision into the membrane closing the external orifice of the vaginal canal, close to the body of the meatus urinarius, with the back of the bistoury towards the meatus. On introducing a silver probe into the incision made, we became satisfied that the vaginal canal was normal, and that the membrane was about two lines in thickness. Then we introduced the directory into the incision made in the membrane, carried it down and backward as far as the fourchette, then divided the membrane on the directory with the probe pointed bistoury. Very little hemorrhage followed the operation. Between the cut surfaces we placed lint moistened with sweet oil, and ordered it to be changed twice in 24 hours.

*May 12th.*—The parts presented a healthy and normal condition.

